# Educational Level, Obesity and Incidence of Diabetes among Chinese Adult Men and Women Aged 18–59 Years Old: An 11-Year Follow-Up Study

**DOI:** 10.1371/journal.pone.0066479

**Published:** 2013-06-20

**Authors:** Xianwen Shang, Jiongyi Li, Qiushan Tao, Jing Li, Xi Li, Lihua Zhang, Xiancheng Liu, Qing Wang, Xiuzhong Shi, Yuhong Zhao, Shuang Hu, Lixin Jiang, Ying Yang

**Affiliations:** 1 State Key Laboratory of Cardiovascular Disease, China Oxford Centre for International Health Research, Fuwai Hospital, National Center for Cardiovascular Diseases, Chinese Academy of Medical Sciences and Peking Union Medical College, Beijing, People’s Republic of China; 2 Qingdao Fuwai Hospital, Qingdao, Shandong, People’s Republic of China; 3 Department of Epidemiology and Biostatistics, School of Public Health, Peking University Health Science Center, Beijing, People’s Republic of China; Istituto Clinico S. Ambrogio, Italy

## Abstract

**Objective:**

To determine whether educational level and overweight/obesity was associated with the development of diabetes among Chinese adult men and women.

**Methods:**

A cohort (2000–2011) of 10 704 participants aged 18–59 years (8 238 men, 2 466 women) in Qingdao Port Health Study (QPHS) were recruited in this study. The personal lifestyle, height, weight, waist circumference, resting heart rate, blood pressure, fasting blood glucose, total cholesterol, triglycerides and plasma uric acid were collected annually in a comprehensive health checkup program. Cox proportional hazards regression models were used to estimate the association of factors and incidence of diabetes.

**Results:**

During 110 825 person-years of follow-up, 1 056 new onset cases (9.5 per 1 000 person-years) of diabetes were identified. With normal weight as reference, the multiple-adjusted hazard ratio (HR) (95%CI) of diabetes was 1.69(1.38–2.09) for overweight and 2.24(1.66–3.02) for obesity among men, which was 1.81(1.12–2.92) and 2.58(1.37–4.86) among women, respectively. Compared with the participants with high educational level, those with low educational level had a higher risk of diabetes (multiple-adjusted HR (95%CI): 1.43(1.11–1.86)) among men. The association was not found among women and the adjusted HR (95%CI) of diabetes was 1.56(0.89–2.76). The increased risks of low educational level were independent of mediators among men, through normal weight (*P* for trend = 0.0313) and overweight (*P* for trend = 0.0212) group but not obesity group (*P* for trend = 0.0957).

**Conclusion:**

Baseline overweight/obesity was an independent risk factor for diabetes for both men and women. Low educational level was adversely associated with incidence of diabetes through normal weight, overweight and obesity groups, with the association being substantially attenuated by mediating factors only in the obesity group among men. The association was not found among women.

## Introduction

The increasing prevalence of diabetes is a major public health problem worldwide, particularly in developing countries. With rapid economic development, nutrition transition, and increasingly sedentary lifestyles, the rate of diabetes is increasing sharply in Asia [1]. The prevalence of diabetes has increased from 0.9% for 1980 to 2.5% for 1994 to 9.7% for 2008 among Chinese population [2]–[3]. It was estimated that about 92 million Chinese adults had diabetes [3], which suggested that China had overtaken India as the global epicenter of the diabetes epidemic [4].

Evidence showed that strong inverse relationship between educational level and the incidence of diabetes was consistent in North America and Europe [5]–[8]. A systematic review found that educational level inequalities in diabetes incidence were more pronounced in women than men in high-income countries [9]. Another systematic review demonstrated that the education inequalities in the risk of diabetes existed in both women and men in Europe [10]. While, there is a strong need for further investigation in middle- and low-income countries [9]. So far, we found no relevant publications from China mainland that has been going through rapid economic development during the three previous decades, together with sharply increasing number of diagnosed diabetes patients. A community cohort study in southern Taiwan revealed that educational level was adversely associated with the development of diabetes (HR = 0.80, 95% CI: 0.60–1.02, *P* value = 0.065), but with no significant difference after controlled for confounding factors [11]. Cross-sectional studies in China mainland showed that educational level was adversely associated with the prevalence of diabetes [2]–[3]. There was no available information on the association of educational level with incidence of diabetes based on longitudinal study in China mainland.

Low educational level may influence diet quality, physical inactivity, and unhealthy behaviors possibly affecting the clustering of diabetes [12]–[13] . Randomized controlled trials demonstrated that lifestyle intervention could reduce the incidence of diabetes, and body mass index (BMI) played an important role in the process [14]–[16]. It was revealed that inverse relationship between educational level and incidence of diabetes is only partially explained by variations in BMI [10]. Our study aims to investigate the association between educational level in combination with overweight/obesity and incidence of diabetes among men and women aged 18–59 years based on an 11-year follow-up study.

## Methods

### Ethical Approval

The study protocol was approved by the Ethics Committee of the Qingdao Fuwai Hospital. Written consents were obtained from all participants.

### The Study Population

The Qingdao Port Health Study cohort was established in 2000 to evaluate potential risk factors for chronic diseases. The study participants consisted of all the employees aged 18 years or more from Qingdao Port Company, which is one of the largest ports in China for international trade and ocean shipping. A total of 12 023 people (Men: 9 227, Women: 2 796) participated in the study at baseline. Information on lifestyle variables, socio-economic status, physical examinations and biomedical variables were collected from each participant annually from 2000 to 2011.

**Figure 1:**
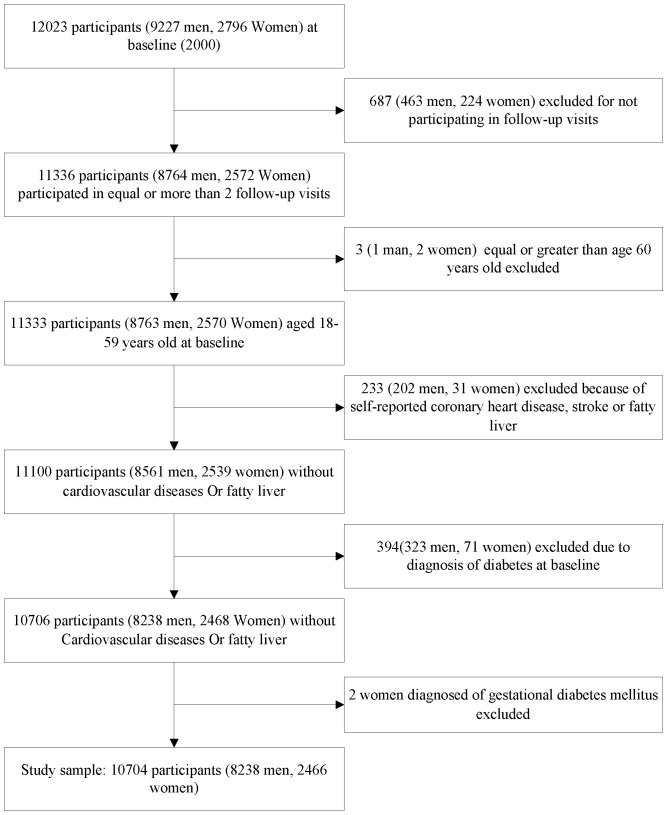
Definition of study participants.

As shown in figure 1, participants with no available information during the 11 subsequent years (n = 687), equal or older than 60 years at baseline (n = 3), those who had been diagnosed with diabetes (n = 394) or coronary heart diseases, stroke or fatty liver at baseline (n = 233), or women cases who got gestational diabetes mellitus (n = 2) were excluded. Consequently, data from 10 704 participants (men 8 238, women 2 466) aged 18–59 years at baseline in 2000 were included in the analysis (Figure 1). Follow-up visits occurred annually during 2001–2011, and a range from 8 741 to 9 446 of participants (return rate: 82%–88%).

### Socio-economic Position

Individual measures of socio-economic position, such as educational level, occupation and household income, were collected with a standardized questionnaire. Educational level was used most frequently to predict the risk of diabetes [10] and it was divided into three levels in our study: low educational level (illiteracy or primary or junior middle school), middle educational level (senior middle/high school) and high educational level (college or above). Occupation was classified as three levels: high grade (higher grade professionals, administrators and officials), middle grade (clerical & admin workers, health services) and low grade (machinery operators, drivers and labourers). Household income (Renminbi) per month was divided into four levels: <500, 500–1199, 1200–1999 and ≥2000.

### Measurements

A self-administered questionnaire was used to collect information about demographics and lifestyle. Covariates at baseline included age (calculated by birthday and the date of physical examination), family history of diabetes (parent and/or sibling had history of diabetes), marriage status (single, married and separated/widowed/devoice), cigarette smoking (never, former and current), alcohol consumption (never, low frequent (1–2 times per week) and high frequent (≥3 times per week)), physical exercise (active (≥1 time per week) and inactive (<1 time per week)), work strength (low, moderate and high), dietary intake (using a simple food frequent questionnaire to evaluate dietary intake) and salt taste preference (light, moderate and salty). Meanwhile, history of diabetes, coronary heart disease, stroke and relative medication was interviewed individually. In addition, participants also received physical examinations, including height, weight, waist circumference (WC), blood pressure (BP) and resting heart rate (RHR) at baseline. Height was measured to the nearest 0.1 cm with a freestanding stadiometer and fasting body weight was measured to the nearest 0.1 kg using a balance-beam scale, with participants wearing lightweight clothing. BMI was calculated as weight in kilogram divided by the square of height in meter (BMI = weight (kg)/(height (meter))^2^). WC was measured midway between the lowest rib and the superior border of the iliac crest. BP was measured three times in the seated position using a column sphygmomanometer with at least 5 minutes rest before the measurement. The averages of the three measures were used in our analysis. RHR was measured using pulse palpation over a 30-s period. Biomedical variables, including fasting plasma glucose (FPG), total cholesterol (TC), triglycerides (TG) and plasma uric acid level (UA), were measured. FPG, TC and TG were measured with an Automatic Analyzer (Hitachi 7060). UA was measured on a standard SMA 12–60 analyzer (Technicon) by the method of Crowley. Information on lifestyle variables, physical examinations and biomedical variables were measured annually for each participant in the subsequent years, except for the dietary consumption, which was only collected in 2000 and 2003. We defined normal weight as BMI<24 kg/m^2^, overweight as BMI 24–28 kg/m^2^, and obesity as BMI≥28 kg/m^2^, according to Working Group on Obesity in China [17].

### Case Ascertainment

Diabetes was defined according to current World Health Organization criteria using fasting concentrations of glucose (FPG ≥7.0 mmol/l at least two different separate investigations) [18] or diabetes for self-reporting or the use of antidiabetic medication at any investigation or diagnosed as diabetes in the medical records. Finally, 1056 cases (men: 911, women: 145) were identified during 11 subsequent years. Given that the youngest participant was diagnosed of diabetes at the age of 21.6 years, most of these cases could be considered as type 2 diabetes [19].

### Statistical Analysis

We compared baseline characteristics of participants from each educational level using chi-square test for categorical variables and ANOVA analysis for continuous variables. Person-years for each participant were calculated from the date of physical examination performed in 2000 to the date of onset diabetes, death, or the end of the follow-up period in 2011, whichever came first. Time-dependent Cox proportional hazards models were used to estimate the HRs of incidence of diabetes related to educational levels and BMI categories. In the multivariate analysis, we adjusted for age, family history of diabetes, marriage status, occupation, cigarette smoking, alcohol consumption, physical exercise, work strength, dietary intake, salt taste preference, WC, RHR, BP, TC, TG, UA and FPG at baseline. Furthermore, we assessed the association longitudinally over the follow-up (2001–2011) and entered variables in the Cox regressions as time dependent variables. The proportional hazard assumptions for Cox regression models were tested to be not violated by using Schoenfeld residuals (all *P* values ≥0.05). It was considered significant if the *P* value <0.05 by two sides. All statistical analyses were done with the SAS 9.2 for Windows (SAS Institute Inc, Cary, NC).

## Results

As shown in table 1, men with low educational level tended to have significantly higher mean baseline FPG and BMI than those with middle educational level (*P*<0.0001 for FPG, *P* = 0.0021 for BMI), while have similar mean baseline FPG in those with high educational level. This tendency did exist in women but without significant difference in FPG (*P* = 0.0832 for FPG, *P*<0.0001 for BMI). And participants with low educational level also have significantly higher age, BP, WC, TC, and TG and lower UA than those with middle and high educational level both in men and women (all *P* values<0.05). In addition, participants with low educational level often consumed more grain, meat and total energy but less fruit than those with high educational level (all *P* values<0.05). There is no significant difference in proportion of family diabetes history between different educational levels for both men and women (*P* = 0.4957 for men, *P* = 0.7489 for women).

**Table 1:** Characteristics of the study groups at baseline in 2000, means ± SD, or N (%).

	Men				Women			
	Low level	Middle level	High level	*P* value	Low level	Middle level	High level	*P* value
N	2529	4324	1385		641	1234	591	
Age (yrs)	43.2±6.6	36.0±9.0	37.7±8.6	<.0001	42.6±4.4	35.4±8.0	38.2±7.8	<.0001
BMI (kg/m^2^)	24.7±3.2	24.4±3.4	24.7±3.3	0.0021	24.2±3.4	22.2±3.0	23.0±3.2	<.0001
BP (mm Hg)								
Systolic	125.4±17.5	120.7±14.3	120.9±14.2	<.0001	113.9±16.3	105.4±13.1	109.4±14.1	<.0001
Diastolic	83.7±12	80.6±10.7	80.9±10.6	<.0001	74.6±10.1	69.4±9.2	71.9±9.4	<.0001
WC (cm)	85.4±9.3	83.8±9.8	84.2±9.5	<.0001	73.9±10.1	69.3±9.1	71.2±9.2	<.0001
RHR (beats/min)	70.9±10.1	71.2±9.5	71.0±9.6	<.0001	72.2±9.8	71.1±9.6	71.9±9.3	0.1319
TC (mmol/L)	4.89±1.05	4.65±1.05	4.65±0.98	0.0009	4.79±1.26	4.41±1.21	4.58±1.17	<.0001
TG (mmol/L)	1.41±1.22	1.30±1.08	1.32±0.88	0.0009	1.08±0.90	0.91±0.67	0.93±0.50	<.0001
UA (umol/L)	225.2±154.1	192.9±163.9	269.3±137.3	<.0001	209.3±114.3	204.2±107.5	217.4±91.9	0.5831
FPG (mmol/L)	5.23±0.84	5.03±0.87	5.17±0.81	<.0001	5.18±1.11	5.06±1.09	5.16±1.08	0.0832
Grain (g/day)	298.1±85.2	302.1±85.3	287.1±84.4	<.0001	193.4±73.9	183.3±62.5	190.4±69.5	0.0312
Meat (g/day)	246.4±121.5	244±123.1	213.8±95.1	<.0001	189.7±110	162.7±77.5	182.5±103.6	<.0001
Vegetable (g/day)	294.9±100.3	295.7±99.1	303.7±99.5	0.0180	262.1±96.7	256.4±92.6	253.9±94	0.1997
Fruit (g/day)	74.9±53.8	84.9±54.3	85.9±54.7	<.0001	104.2±61.7	120.1±56.3	117±60.1	<.0001
Total energy intake (kcal/day)	2035.9±527.4	2046.4±544.2	1894.4±486.3	<.0001	1476.1±509.5	1442.1±458.1	1354.5±363.4	<.0001
Family History	302(12.4)	486(11.9)	183(13.2)	0.4957	84(13.1)	152(12.4)	74(12.6)	0.7489
Working Strength								
Light	58(2.3)	419(9.7)	729(52.6)	<.0001	36(5.6)	386(31.6)	434(73.9)	<.0001
Moderate	1857(73.5)	3367(77.9)	610(44.0)		572(89.4)	825(67.5)	152(25.9)	
High	611(24.2)	538(12.4)	46(3.3)		32(5.0)	11(0.9)	1(0.2)	
Physical Exercise	1777(70.3)	2637(61.0)	840(60.6)	<.0001	524(81.7)	907(74.2)	407(69.3)	<.0001
Salt preference								
Salty	595(23.5)	776(17.9)	294(21.2)	0.0006	92(14.4)	159(13)	67(11.4)	0.0837
Medium	1732(68.5)	3160(73.1)	961(69.4)		486(75.8)	946(77.4)	451(76.8)	
Not salty	202(8.0)	388(9.0)	130(9.4)		63(9.8)	117(9.6)	69(11.8)	
Former smoker/Current smoker	2071(81.9)	3015(69.8)	827(59.8)	<.0001	5(0.8)	3(0.2)	0(0)	0.4602
Alcohol intake								
Never	716(28.3)	1525(35.3)	462(33.4)	<.0001	624(97.3)	1194(97.7)	571(97.3)	<.0001
Low frequent	785(31.0)	1828(42.3)	656(47.4)		7(1.1)	19(1.6)	15(2.6)	
High frequent	1028(40.6)	971(22.5)	267(19.3)		10(1.6)	9(0.7)	1(0.2)	
Occupation								
Low grade	2206(87.2)	3359(77.7)	465(33.6)	<.0001	428(66.8)	686(56.1)	123(21.0)	<.0001
Middle grade	279(11.0)	474(11.0)	129(9.3)		200(31.2)	259(21.2)	73(12.4)	
High grade	44(1.7)	491(11.4)	791(57.1)		13(2.0)	277(22.7)	391(66.6)	
Marriage status								
Single	84(3.3)	962(22.2)	186(13.4)	<.0001	5(0.8)	114(9.3)	83(14.1)	<.0001
Married	2401(94.9)	3320(76.8)	1193(86.1)		602(93.9)	1070(87.6)	490(83.5)	
Separated/Widowed/Devoice	44(1.7)	42(1.0)	6(0.4)		34(5.3)	38(3.1)	14(2.4)	

Educational level differences were compared using chi-square test for categorical variables and ANOVA analysis for continuous variables.

Age, occupation, cigarette smoking, alcohol consumption, physical exercise, BMI, WC, RHR, BP, TC, TG, UA, and FPG were collected annually from 2000 to 2011.

Family history of diabetes, marriage status, and work strength were collected annually from 2000 to 2003.

Dietary intake and salt taste preference were collected in 2000 and 2003.

**Figure 2:**
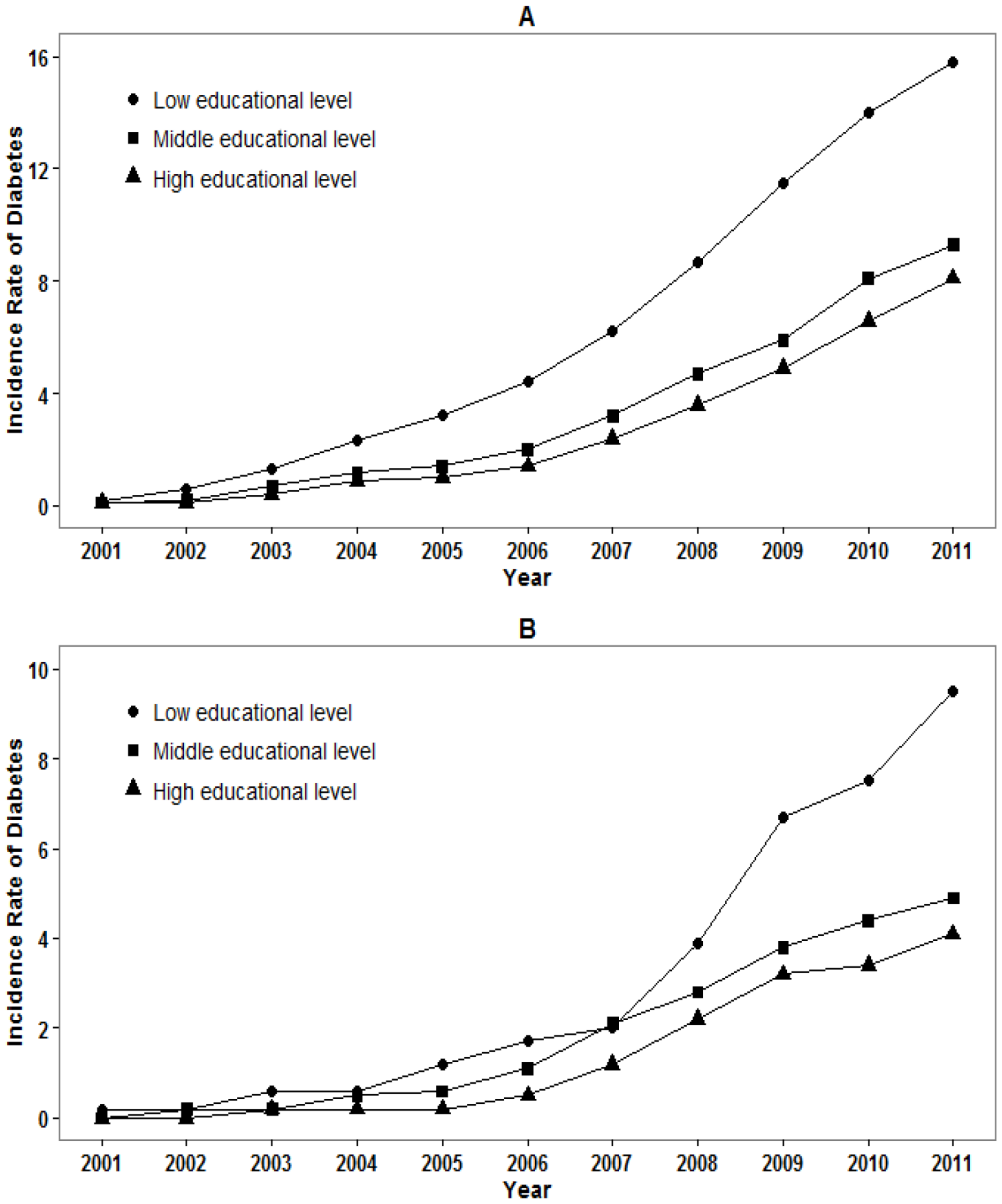
Cumulative incidence (%) of diabetes among men (A) and women (B) by educational level.

During 110 825 person-years of follow-up, 1 056 new onset cases (9.5 per 1 000 person-years) of diabetes were identified. The slope of cumulative incidence of type 2 diabetes among men with low educational level was much higher than that of middle and high educational level. The cumulative incidence in 2011 was 15.8%, 9.3% and 8.1% for those with low, middle and high educational level, respectively (Figure 2(A)). The cumulative incidence showed a similar tendency among women and up to 9.5%, 4.5% and 4.1% in 2011 for those with low, middle and high educational level, respectively (Figure 2(B)).

There was a significant interaction between educational level and gender with incidence of diabetes (*P* = 0.0097). The incidence of diabetes of men was much higher than that of women (men: 11.0%, women: 6.2%, *P*<0.0001). Men with low educational level (HR (95% CI): 1.69(1.31–2.19)) showed a higher risk of diabetes than those with high educational level, independent of family history of diabetes, baseline age, marriage status, occupation, cigarette smoking, alcohol consumption, physical exercise, work strength, dietary intake and salt taste preference. After multivariable adjustment, the association was still significant (HR (95% CI): 1.53(1.22–1.93)). In longitudinal Cox regressions model, after adjustment for baseline variables and time dependent variables, low educational level was still a risk factor for incidence of diabetes (HR (95% CI): 1.43(1.11–1.86)). The association of educational level with incidence of diabetes was also found among women (HR (95%CI) for low level vs. high level: 2.51(1.57–4.02), middle level vs. high level: 1.26(0.79–2.02)). After adjustment for family history of diabetes, baseline age, marriage status, occupation, cigarette smoking, alcohol consumption, physical exercise, work strength, dietary intake and salt taste preference, the association attenuated with no significant difference among women (HR (95%CI) for low level vs. high level: 1.56(0.89–2.76), HR (95%CI) for middle level vs. high level: 1.02(0.60–1.75)). Overweight and obesity was positively associated with the incidence of diabetes (HR (95%CI) for overweight vs. normal: 2.60(2.19–3.08), HR (95%CI) for obesity vs. normal: 4.73(3.94–5.68)) among men. The association attenuated in the longitudinal model, but remained still significant (HR (95%CI) for overweight vs. normal: 1.69(1.38–2.09), HR (95%CI) for obesity vs. normal: 2.24(1.66–3.02)). The hazard ratio for overweight and obesity versus the normal weight among women was 4.35(2.94–6.44) and 9.12(5.95–14.23), respectively. This attenuated to 2.57(1.64–4.02) and 3.73(2.04–6.84) when we controlled for multivariable and 1.81(1.12–2.92) and 2.58(1.37–4.86) when time-dependent variables were accounted for (as shown in Table 2).

**Table 2:** Hazard ratios for incidence of diabetes among men and women.

	Model 1	Model 2	Model 3	Model 4
**Men**				
Educational Level*				
High	1.00	1.00	1.00	1.00
Middle	1.16(0.94–1.44)	1.34(1.06–1.69)	1.23(1.00–1.53)	1.17(0.93–1.47)
Low	2.05(1.66–2.53)	1.69(1.31–2.19)	1.53(1.22–1.93)	1.43(1.11–1.86)
* P* for trend	<.0001	0.0005	0.0179	0.0207
BMI category†				
Normal	1.00	1.00	1.00	1.00
Overweight	2.60(2.19–3.08)	2.21(1.86–2.62)	1.64(1.35–1.98)	1.69(1.38–2.09)
Obese	4.73(3.94–5.68)	3.99(3.31–4.81)	2.25(1.76–2.89)	2.24(1.66–3.02)
* P* for trend	<.0001	<.0001	<.0001	0.0015
**Women**				
Educational Level*				
High	1.00	1.00	1.00	1.00
Middle	1.26(0.79–2.02)	1.02(0.60–1.75)	0.87(0.54–1.39)	0.86(0.49–1.51)
Low	2.51(1.57–4.02)	1.56(0.89–2.76)	1.06(0.65–1.74)	1.15(0.60–2.21)
* P* for trend	0.0354	0.3636	0.4046	0.6115
BMI category†				
Normal	1.00	1.00	1.00	1.00
Overweight	4.35(2.94–6.44)	3.25(2.17–4.87)	2.57(1.64–4.02)	1.81(1.12–2.92)
Obese	9.12(5.95–14.23)	6.63(4.18–10.51)	3.73(2.04–6.84)	2.58(1.37–4.86)
* P* for trend	<.0001	<.0001	0.0049	0.0082

Model 1: unadjusted. Model 2: adjusted for family history of diabetes, baseline age, marriage status, occupation, cigarette smoking, alcohol consumption, physical exercise, work strength, dietary intake and salt taste preference. Model 3: adjustments in model 2 plus baseline WC, RHR, BP, TC, TG, UA and FPG. Model 4: adjustments for risk factors in model 3 as time dependent variables.

* Added BMI category in model 3 when accessing the association of educational level with incidence of diabetes.

† Added educational level in model 2 when accessing the association of overweight and obesity with incidence of diabetes.

There was a significant interaction between educational level and overweight/obesity with incidence of diabetes among men (*P* = 0.0201). Low educational level was inversely associated with the incidence of diabetes among men, through normal weight, overweight group but not obesity group (*P* for trend = 0.0313 for normal, *P* for trend = 0.0212 for overweight, *P* for trend = 0.0957 for obesity), when long term exposure is accounted for. The association was found among women in normal weight group (*P* for trend = 0.0030), which was attenuated when controlling for lifestyle variables (*P* for trend = 0.1182). The association was not found in other groups among women (*P* for trend = 0.7952 for overweight, *P* for trend = 0.7049 for obesity), as shown in table 3. And interaction between educational level and overweight/obesity with incidence of diabetes was not significant among women (*P* = 0.0798).

**Table 3:** Association between educational level and incidence of diabetes among men and women at each BMI category.

	Normal		Overweight		Obesity	
	HR(95%CI)	*P* for trend	HR(95%CI)	*P* for trend	HR(95%CI)	*P* for trend
**Men**						
Model 1	0.68(0.60–0.77)	<.0001	0.80(0.73–0.87)	<.0001	0.81(0.73–0.90)	<.0001
Model 2	0.82(0.72–0.93)	0.0018	0.87(0.8–0.95)	0.001	0.86(0.78–0.96)	0.0068
Model 3	0.81(0.70–0.93)	0.0040	0.88(0.8–0.97)	0.01	0.86(0.76–0.97)	0.0132
Model 4	0.83(0.73–0.95)	0.0064	0.9(0.82–0.98)	0.013	0.91(0.81–1.01)	0.0734
Model 5	0.85(0.74–0.99)	0.0313	0.89(0.81–0.98)	0.021	0.90(0.79–1.02)	0.0957
**Women**						
Model 1	0.57(0.43–0.74)	<.0001	0.99(0.81–1.22)	0.937	0.93(0.71–1.21)	0.5707
Model 2	0.67(0.51–0.89)	0.0061	1.02(0.83–1.26)	0.827	0.99(0.76–1.29)	0.9413
Model 3	0.65(0.49–0.87)	0.0036	1.04(0.81–1.34)	0.762	0.95(0.7–1.31)	0.7726
Model 4	0.76(0.55–1.05)	0.0954	1(0.81–1.24)	0.994	1(0.75–1.33)	0.9736
Model 5	0.76(0.53–1.07)	0.1182	0.97(0.74–1.27)	0.795	1.07(0.75–1.52)	0.7049

Model 1: unadjusted. Model 2: adjusted for baseline age, family history of diabetes, marriage status and occupation. Model 3: adjustments in model 2 plus baseline cigarette smoking, alcohol consumption, physical exercise, work strength, dietary intake and salt taste preference. Model 4: adjustments in model 3 plus baseline WC, RHR, BP, TC, TG, UA and FPG. Model 5: adjustments for risk factors in model 4 as time dependent variables.

### Sensitivity Analysis

We repeated all analyses in subgroups, including participants with 10 or 11 follow-up visits, participants who had information on household income, participants who had full records of weight and height at last visit in 2011, or participants who provided information on weight at 25 years.

To determine whether missing visits could change the association, we repeated the analyses among participants (5 495 men, 1 519 women) who returned 10 or 11 visits. Results were similar with those in the major analyses. Men with low educational level (HR: 1.44, 95% CI: 1.05–1.98, *P* = 0.0280) showed a higher risk of diabetes incidence than those with high educational level, when long term exposure to risk factors were accounted for. The association was still not found among women. And overweight/obesity was still significantly associated with incidence of diabetes for both men and women.

Furthermore, we repeated the analyses among participants (4 729 men, 1 939 women) who provided information about household income. With the high educational level as reference, the hazard ratio (95% CI) of diabetes for low educational level changed from 1.42(1.02–1.97) to 1.42(1.02–1.98), after further adjustment of household income based on model 4. Household income did not change the association of incidence of diabetes with educational level and overweight/obesity among women.

Thirdly, we included the attained BMI as a mediator in the Cox model among participants (6 705 men, 2 036 women) who had full records of weight and height at last visit in 2011. The association of educational level with diabetes incidence was similar to that reported in main analysis. And baseline overweight/obesity was still an independent predictor for the development of diabetes for both men and women.

Finally, we repeated the analyses among participants (7 653 men, 2 271 women) who provided information about the weight at 25 years old. The results were similar before and after adjustment of weight at 25 years old for both men and women when we performed the analysis in all the models.

## Discussion

In the present study, educational level was adversely associated with the incidence of diabetes among men, while the association was eliminated among women when lifestyle factors were accessed at baseline. Baseline overweight/obesity was an independent predictor for the development of diabetes for both men and women, regardless of the changes in lifestyle variables, physical examinations and biomedical variables during 11 subsequent years. Our results also suggested that there was a significant interaction between educational level and overweight/obesity in predicting diabetes among men, with low educational level showing a higher risk at normal and overweight groups that decreased by obesity group.

Study showed that there is a considerable burden of diabetes attributed to lower educational levels for both men and women in Sweden [20]. In China mainland, only cross-sectional studies reported the adverse relationship of educational level and the prevalence of diabetes [2]–[3]. In the present study, it was revealed that educational level was an independent predictor for the incidence of diabetes among men but not among women. After adjustment of baseline physical examinations and biomedical variables, the HR (95% CI) of diabetes for low educational level among men, with high educational level as reference, changed from 1.69(1.31–2.19) to 1.55(1.29–1.93). And the HR (95% CI) lessened to 1.44(1.12–1.86), when considering lifestyle variables and metabolic factors as time-dependent variables. The association between educational level and the incidence of diabetes among women was not significant, after adjustment of baseline lifestyle factors. Our study was consistent with Robbins’s study, which showed that mediating factors eliminated the risk of incidence of diabetes with education among women, but was not substantially attenuated by mediate factors among men [21]. The Whitehall II study with data from an occupational cohort indicated that socioeconomic gradient in incidence of diabetes did not differ by sex [22]. The sex differences observed may be due to low incidence of diabetes and smaller number of women included in our study.

The major role of excess weight in the development of diabetes is well established [23]. A prospective study between 1983 and 1994 indicated that the increase in incidence of diabetes associated with one-unit increase in BMI was 1.7 percentage points among Chinese, which was similar with that observed in Blacks, but higher than that in Whites [24]. Liu et al. examined the risk factors of diabetes and found that overweight and obesity defined by BMI were predictors for diabetes using a prospective data between 1999 and 2004 [25]. Our study investigated the association of overweight and obesity with cumulative incidence of diabetes based on a longitudinal data between 2000 and 2011, which revealed that overweight/obesity was an independent predictor of diabetes for both men and women. The hazard ratio of diabetes for overweight (vs. normal) and obesity (vs. normal) were attenuated both among men and women, after adjustment of physical examinations and biomedical variables at baseline, which was further attenuated among women but not among men, when additionally considering lifestyle variables and metabolic factors as time-dependent variables. So, our study indicated that the relation between overweight/obesity and the incidence of diabetes could be explained evidently by baseline metabolic factors for both men and women, and further could be explained much by lifestyle variables and metabolic factors changes in subsequent years among women but not among men.

We also determined the interaction of educational level and overweight/obesity in predicting the incidence of diabetes. Among men, educational level was adversely associated with the incidence of diabetes in normal weight, overweight and obesity group. The association was attenuated only in the obesity group, when controlling metabolic factors, such as baseline WC, RHR, BP, TC, TG and UA. Participants with high educational level in our study also had healthier behaviors and low risk of hyperlipemia, which might have preventive effects on diabetes [26]–[28]. When all these mediating factors were controlled, the association of educational level and diabetes could not still be explained in the normal weight and overweight groups. The association of educational level and the incidence of diabetes was only found in normal weight group among women (*P* for trend = 0.006), which attenuated with no significant difference (*P* for trend = 0.0954) when metabolic factors were accounted for. Consistent with previous studies, metabolic factors could be considered as risk factors for the development of diabetes [29]–[30].

The major strength of the present study is the long follow-up period with high return rate in a population-based sample. The major limitation of the present study is that there was much higher proportion of men than women in our sample. It has long been considered that diet played a role in the onset of diabetes [31]–[32]. Dietary intake was only collected in 2000 and 2003 in our study, which may underestimate the effect of diet on diabetes. There are still some other mediating factors of the association of educational level and diabetes unavailable in our study, such as birth weight [33] and exposure to adverse socioeconomic environments in childhood [34]. Further studies should collect additional information on birth weight, diet and parents’ socioeconomic positions to determine whether the association of educational level and incidence of diabetes can be explained by these factors.

### Conclusion

Baseline overweight/obesity was an independent risk factor of diabetes for both men and women even when the change of lifestyle variables, physical measurements, and biomedical variables during the follow-up period were accounted for. Educational level was adversely associated with incidence of diabetes through normal weight, overweight and obesity groups, with the association being substantially attenuated by metabolic factors only in the obesity group among men. The association was not found among women.
